# The phased *Solanum okadae* genome and *Petota* pangenome analysis of 23 other potato wild relatives and hybrids

**DOI:** 10.1038/s41597-024-03300-5

**Published:** 2024-05-04

**Authors:** S. R. Achakkagari, I. Bozan, J. C. Camargo-Tavares, H. J. McCoy, L. Portal, J. Soto, B. Bizimungu, N. L. Anglin, N. Manrique-Carpintero, H. Lindqvist-Kreuze, H. H. Tai, M. V. Strömvik

**Affiliations:** 1 Department of Plant Science, McGill University, Sainte-Anne-de-Bellevue, QC Canada; 2 Department of Chemistry, University of New Brunswick, Fredericton, NB Canada; 3 International Potato Center (CIP), Lima, Peru; 4 Agriculture and Agri-Food Canada Fredericton Research and Development Centre, Fredericton, NB Canada; 5 USDA ARS Small Grains and Potato Germplasm Research, Aberdeen, ID USA; 6 Present Address: Alliance of Bioversity International and International Center for Tropical Agriculture (CIAT), Cali, Colombia

**Keywords:** Plant genetics, Computational biology and bioinformatics

## Abstract

Potato is an important crop in the genus *Solanum* section *Petota*. Potatoes are susceptible to multiple abiotic and biotic stresses and have undergone constant improvement through breeding programs worldwide. Introgression of wild relatives from section *Petota* with potato is used as a strategy to enhance the diversity of potato germplasm. The current dataset contributes a phased genome assembly for diploid *S. okadae*, and short read sequences and *de novo* assemblies for the genomes of 16 additional wild diploid species in section *Petota* that were noted for stress resistance and were of interest to potato breeders. Genome sequence data for three additional genomes representing polyploid hybrids with cultivated potato, and an additional genome from non-tuberizing *S. etuberosum*, which is outside of section *Petota*, were also included. High quality short reads assemblies were achieved with genome sizes ranging from 575 to 795 Mbp and annotations were performed utilizing transcriptome sequence data. Genomes were compared for presence/absence of genes and phylogenetic analyses were carried out using plastome and nuclear sequences.

## Background & Summary

Potato is currently the third most important crop for human consumption with increasing production in the developing world and it is an important sustainable global food security crop for climate-smart agriculture^1,2^. Breeding for improved nutritious potato varieties suitable for sustainable production and climate change resilience will be aided by introgression of diverse genes from native Andean cultivars and landraces along with wild *Solanum* relatives of potato^3^. The genebank at the International Potato Center (CIP) and other genebanks globally, carry diverse germplasm used by breeding programs for genetic improvement of the crop^4,5^. The CIP genebank has potato cultivars, landraces, and wild *Solanum* germplasm collected from a wide range of environments in North and South America with variation in abiotic and biotic stress. Native Andean cultivars and landraces also carry beneficial nutrients and bioactive compounds for human health^6,7^.

A reference genome sequence for a doubled monoploid potato clone DM1-3 516 R44 (DM) was released in 2011 and later improved^8–10^. Low depth sequencing of potato varieties and wild species aligned against the DM reference was used to assess genome diversity, copy number variation (CNVs), introgressions, and selective sweeps^11–13^. Since then, the genomes of additional potato clones, diploid as well as tetraploid, have been sequenced and *de novo* assembled^14–20^.

Potato and its wild relatives are in the genus *Solanum* section *Petota*, which consists of over 100 species that form tubers^21^. Increasing knowledge on the genetic potential of species in section *Petota* will increase capacity for their use in enhancing potato germplasm. Genomes of several wild species are also available^22–25^ and a *Petota* super pangenome representing 60 species was recently released^26^. The current data set includes a phased genome assembly of the wild species *S. okadae*, for which short reads and the mitogenome were previously published^26,27^. In addition, the study contributes Illumina short read sequences (50–100x sequencing depth) and *de novo* assemblies (3,328–59,223 N50) for genomes of 16 wild species in section *Petota* that were noted for stress resistance and of interest to potato breeders. An *S. etuberosum* genome from outside section *Petota* was also included in this data set, as were three polyploid hybrids. For most of the species in this project, these are the first genome sequences released of their species. Taxonomic groupings of the species of section *Petota* into Clade 1 + 2, Clade 3 and Clade 4 were represented in the genomes sequenced.

Transcriptome sequence data was generated and used for annotation. Both nuclear and organellar genome sequences are provided. Comparative analysis of the presence/absence variation against the *Petota* super pangenome provided insight into the diversity of the species in the phylogenetic analysis.

## Methods

### *Solanum okadae* (OKA15) genome sequencing

Botanical *S. okadae* seed sourced from US Genebank NRSP-6 (PI 458367) were sown on agar media. Individual clones were propagated *in vitro*. Seventeen lines were planted in the greenhouse and self-pollinated. While abundant flowering was noted, a single line, OKA15, was selected for further study^26,27^. The 10X Genomics short reads were previously published^26,27^. High molecular weight DNA for *S. okadae* OKA15 was prepared using a modified CTAB procedure^28^ and used for Hi-C (chromatin interactions), Pacific Biosciences (PacBio) and Nanopore sequencing. The Hi-C library preparation was done using Phase Genomics Proximo Plant Hi-C version 4.0, and sequenced at Génome Québec’s Sequencing centre, Montreal, Canada, on Illumina NovaSeq. 6000 platform in PE 100 bp mode with ~3B reads, while both the PacBio libraries (sequenced on Sequel II system using the SMRT technology (HiFi) at a depth of 100X,) and the Nanopore ONT libraries (sequenced on the PromethION at a depth of 100X) were prepared and sequenced by Novogene (Novogene Co., Ltd, Beijing, China).

### Genome and transcriptome sequencing

A total of 23 additional accessions representing potato wild relatives or hybrids with agronomically important traits such as disease resistance, cold and drought tolerance, and other biotic and abiotic stresses were selected for this study (Table 1). The genomic DNA of these accessions was isolated from *in vitro* or greenhouse grown plantlets or leaves. The library construction, quality control, and sequencing were done by Novogene (Novogene Co., Ltd, Beijing, China). The genomic DNA was fragmented, ligated with adapters, and PCR amplified to construct single libraries with an insert size of 350 bps. The sequencing was performed on the Illumina NovaSeq. 6000 platform, in a paired-end mode (2 X 150 bp) at ~50x or ~100x depth (Table 2). Similarly, total RNA was extracted from these accessions, including from OKA15, from leaf, shoot, flower, and/or tuber tissues. The RNA-Seq library construction used standard protocol (Novogene) and sequencing was done on the Illumina NovaSeq. 6000 platform (PE150) (Table 2).

**Table 1:** A list of the 24 potato wild relatives (*Solanum* sp) accessions selected for this study with their detailed descriptions.

Short ID	Species (Taxonomic group, Spooner)	CIP/ID number	other ID	ploidy	Source location	Important traits
oka15	*S. okadae* (Clade 4)	—	—	2n = 24	AAFC Fredericton- CPGR	resistance: CPB, moderate late blight; drought, cold tolerance
B1595106	*S. brevicaule ( = S. oplocense) X S. tuberosum* (Clade 4)	PGR-15951-06	—	2n = 48	AAFC Fredericton- CPGR	*S. brevicaule* ( = *S. oplocense*) X *S. tuberosum* backcross clone; resistance: CPB
blv1353	*S. boliviense* (Clade 4)	CIP761353.009	—	2n = 24	CIP	drought tolerance
buk0368	*S. bukasovii* (Clade 4)	CIP760368.015	—	2n = 24	CIP	drought tolerance
chc0917	*S. chacoense* (Clade 4)	CIP760917.1	PI 197760	2n = 24	CIP	resistance: bacterial wilt
chq2573	*S. chiquidenum* (Clade 3)	CIP762573.219	OCHS 12566	2n = 24	CIP	resistance: late blight
cmm1080	*S. commersonii* (Clade 4)	CIP761080.201	—	2n = 24	CIP	resistance: bacterial wilt
cxa70P5	*S. commersonii X S. andigena* (Clade 4)	H6S170P5	—	2n = 48	CIP	cultivar- Winay; frost tolerant variety
etb0161	*S. etuberosum* (outgroup)	—	PI 245939	2n = 24	USDA Aberdeen, ID	resistance: insect, PLRV; cold tolerance, self compatible
grc1498	*S. gracilifrons* (Clade 4)	CIP761498.010	—	2n = 24	CIP	drought tolerance
ifd1359	*S. megistacrolobum* (Clade 4)	CIP761359.032	—	2n = 24	CIP	drought tolerance
lgl2830	*S. lignicaule* (Clade 4)	CIP762830.057	—	2n = 24	CIP	drought tolerance
mga1403	*S. megistacrolobum* (Clade 4)	CIP761403.208	OCH 12032	2n = 24	CIP	resistance: bacterial wilt
pcs2126	*S. paucissectum* (Clade 3)	CIP762126.217	OCHS 14818	2n = 24	CIP	resistance: late blight, CPB
pur1868	*S. piurae* (Clade 3)	CIP761868.202	OCH 13959.6	2n = 24	CIP	resistance: late blight, CPB
S12	*S. tarijense* (Clade 4)	DPM-S12	PI 473243	2n = 24	AAFC Fredericton- CPGR	secretory trichome A hairs; resistance: peach aphid, CPB
S15	*S. gandarillasii* (Clade 4)	DPM-S15	PI 545864	2n = 24	AAFC Fredericton- CPGR	drought tolerance
S3	*S. commersonii* (Clade 4)	DPM-S3	PI 472837	2n = 24	AAFC Fredericton- CPGR	leaf roll susceptible, cold tolerance
S7	*S. pinnatisectum* (Clade 1 + 2)	DPM-S7	PI 275236	2n = 24	AAFC Fredericton- CPGR	resistance: insect, PVY; PVX susceptible, Verticillium wilt susceptible, frost susceptible, drought tolerance
SH7_18_3	*S. tarnii X S. tuberosum* (Clade 1 + 2/4)	PGR-7/18/3	—	2n = 48	AAFC Fredericton- CPGR	F1 *tarnii* somatic hybrid (*S. tarnii x tbr* (4x))
spl0147	*S. sparsipilum* (Clade 4)	CIP760147.7	PI 1230502	2n = 24	CIP	resistance: bacterial wilt
tcn8662	*S. tacnaense* (Clade 4)	CIP762866.026	—	2n = 24	CIP	drought tolerance
tcn8663	*S. tacnaense* (Clade 4)	CIP762866.038	—	2n = 24	CIP	drought tolerance
trp2833	*S. tarapatanum* (Clade 4)	CIP762833.025	—	2n = 24	CIP	drought tolerance

**Table 2:** Details of the tissues used and amount of data generated for WGS and RNA-Seq. The raw data is calculated as: number of reads*sequence length (150 bp).

Short ID	Species	Sequencing mode	WGS			RNA-seq	
			Tissue	Sequencing depth (X)	Raw data (Gbp)	Tissues	Raw data (Gbp)
oka15	*S. okadae*	PE150/10X	Leaf	100	66.2	—	—
oka15	*S. okadae*	PacBio	Leaf	100	64.6	—	—
oka15	*S. okadae*	Nanopore	Leaf	100	60.3	—	—
oka15	*S. okadae*	Hi-C	Leaf	—	300	—	—
oka15	*S. okadae*	PE150	Leaf	100	—	Leaf, Tuber, Sprout	35.9
B1595106	*S. brevicaule* ( = *S. oplocense*) *X S. tuberosum*	PE150	Leaf	50	41.5	—	—
blv1353	*S. boliviense*	PE150	Leaf	100	81.7	Leaf	9
buk0368	*S. bukasovii*	PE150	Leaf	100	89.5	Leaf	7.1
chc0917	*S. chacoense*	PE150	*In vitro* plantlets	100	81.5	Leaf, Shoot, Flower, Tuber	68.9
chq2573	*S. chiquidenum*	PE150	*In vitro* plantlets	100	92	Leaf, Shoot	45.3
cmm1080	*S. commersonii*	PE150	*In vitro* plantlets	50	43.5	Leaf, Shoot, Tuber	53.1
cxa70P5	*S. commersonii X S. andigena*	PE150	Leaf	50	40.3	Shoot	6.4
etb0161	*S. etuberosum*	PE150	Leaf	100	80.3	Leaf	9.2
grc1498	*S. gracilifrons*	PE150	Leaf	50	41.2	Leaf	7.5
ifd1359	*S. megistacrolobum*	PE150	Leaf	100	88.1	Leaf	7.8
lgl2830	*S. lignicaule*	PE150	Leaf	100	84.1	Leaf	8.7
mga1403	*S. megistacrolobum*	PE150	*In vitro* plantlets	100	82.5	Leaf, Shoot, Flower	54.5
pcs2126	*S. paucissectum*	PE150	*In vitro* plantlets	100	80.7	Leaf, Shoot	48.7
pur1868	*S. piurae*	PE150	*In vitro* plantlets	100	80.3	Leaf, Shoot	50.8
S12	*S. tarijense*	PE150	Leaf	100	82.9	Leaf, Tuber	13.2
S15	*S. gandarillasii*	PE150	Leaf	100	84.9	Leaf, Tuber	20.8
S3	*S. commersonii*	PE150	Leaf	50	44.8	Leaf, Tuber	14.4
S7	*S. pinnatisectum*	PE150	Leaf	100	80.8	Leaf	21.9
SH7_18_3	*S. tarnii X S. tuberosum*	PE150	Leaf	50	41.6	—	—
spl0147	*S. sparsipilum*	PE150	*In vitro* plantlets	100	88.7	Leaf, Shoot	46
tcn8662	*S. tacnaense*	PE150	Leaf	100	80.4	Leaf	8
tcn8663	*S. tacnaense*	PE150	Leaf	100	80.3	Leaf	8.7
trp2833	*S. tarapatanum*	PE150	Leaf	50	40.3	Leaf, Shoot, Flower	53.2

### *S. okadae* OKA15 phased genome assembly and quality control

**Fig. 1:**
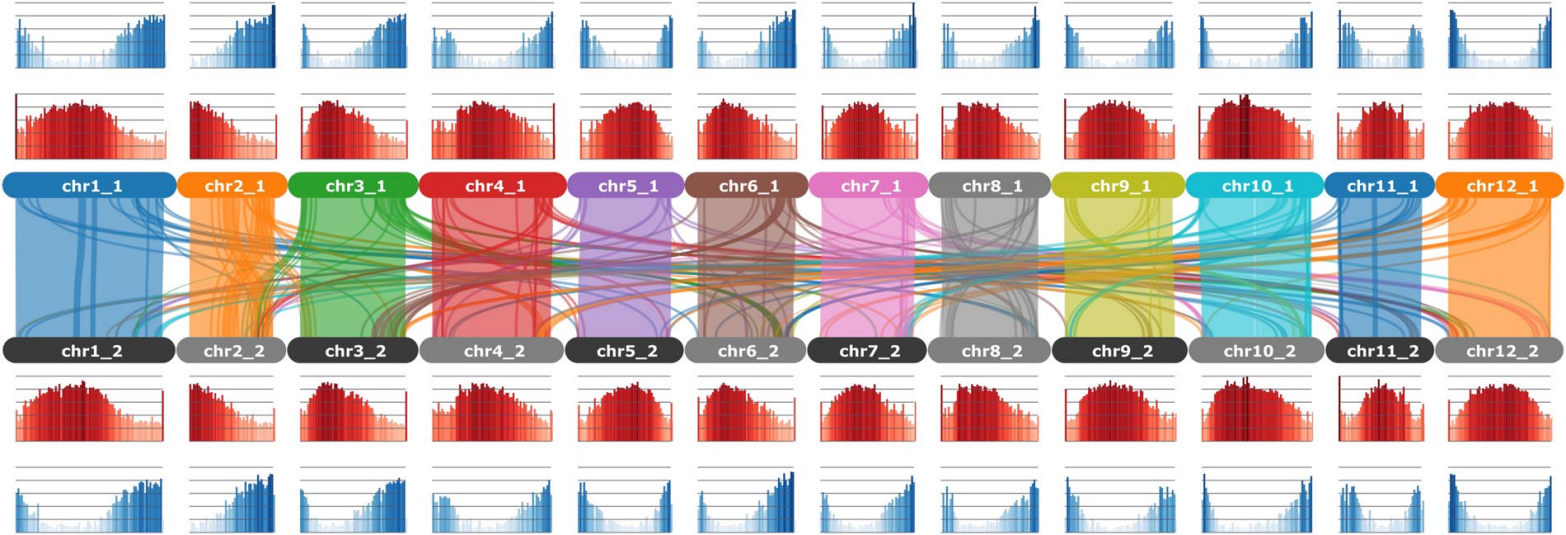
The haplotypes of the phased *Solanum okadae* (OKA15) genome.

The Nanopore reads were adapter trimmed using *porechop v0.2.4* (https://github.com/rrwick/Porechop) and default parameters. *hifiasm v0.16.1-r375*^29^ was used to generate a haplotype-resolved assembly using the PacBio HiFi and the Hi-C reads (Fig. 1). Organellar genomes were removed from both resulting haplotype assemblies. The HiFi and Nanopore reads were used to scaffold the haplotype assemblies. Hi-C reads were trimmed using *fastp v0.23.1*^30^, and mapped to haplotype assemblies individually using the Arima mapping pipeline (https://github.com/ArimaGenomics/mapping_pipeline) and default parameters. The alignments were filtered to keep only the unique alignments (*samtools view* -q 40)^31^. The scaffolding was performed using *YaHS*^32^. Hi-C maps were generated using *Juicer*^33^ and manually reviewed using *JBAT*^33^. Any duplicated contigs from the assemblies were removed using mummer^34^. Gaps were closed using *tgsgapcloser*^35^ with PacBio and Nanopore reads. A custom repeat library was constructed using *Repeatmodeler*^36^ and repeats were masked using *Repeatmasker*^37^ and default parameters. RNA-Seq reads were filtered using *Kraken2*^38^ and trimmed using *fastp*^30^, then aligned to the assembly using *hisat2*^39^. Structural annotations were identified using *Braker3*^40^, and curated using *gfacs*^41^, and functional annotations were generated using *ahrd* (https://github.com/groupschoof/AHRD) and *interproscan*^42^.

### Quality control and analysis of WGS and RNA-Seq for additional potato wild relatives

**Fig. 2:**
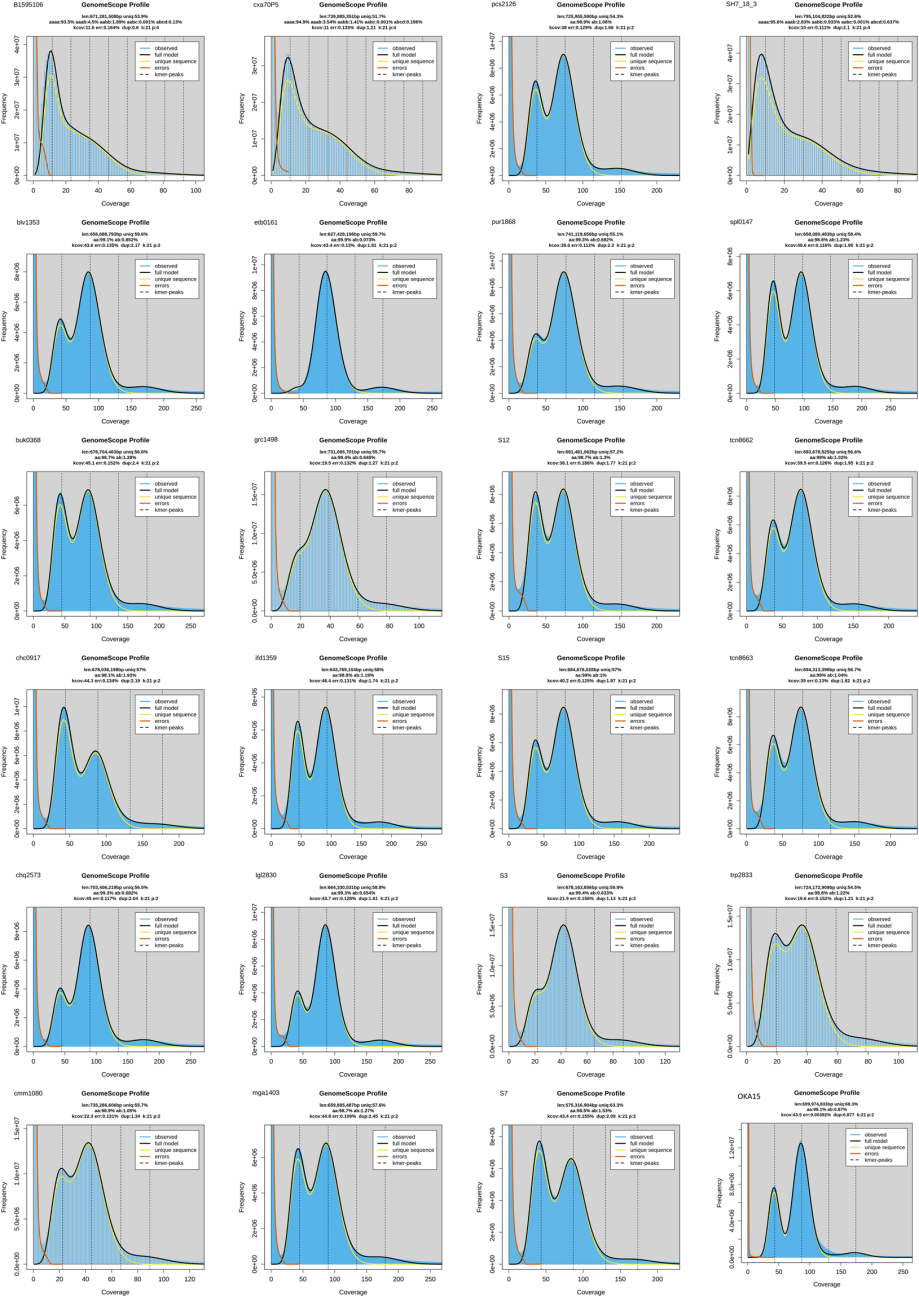
The k-mer coverage plots of each of 23 genome sequences from potato wild relatives (*Solanum* sp).

Quality control was performed on the raw sequencing data (WGS and RNA-Seq) using *FastQC v0.11.9*^43^. An adaptor removal and read quality trimming was performed on the raw reads using *Trimmomatic v0.39*^44^. Genome heterozygosity and estimated size were determined using the trimmed WGS reads. First, k-mer frequencies were calculated for each genome (k = 21) and a k-mer histogram was generated using *Jellyfish v2.3.0*^45^. The k-mer histogram was used to generate various genome characteristics with *GenomeScope 2.0*^46^. The polyploid genomes of *S. brevicaule* (=*oplocense*) *X S. tuberosum* (B1595106), *S. tarnii X S. tuberosum* (SH7_18_3), and *S. commersonii X S. andigena* (cxa70P5) hybrids showed higher rates of heterozygosity (Fig. 2), as seen in previous potato polyploid genomes^20^, while the *S. etuberosum* (etb0161) genome showed very low levels of heterozygosity. The estimated haploid genome sizes ranged from 575 Mbp in *S. pinnatisectum* (S7) to 795 Mbp in *S. tarnii X S. tuberosum* (SH7_18_3).

### *De novo* assembly and annotation

**Fig. 3:**
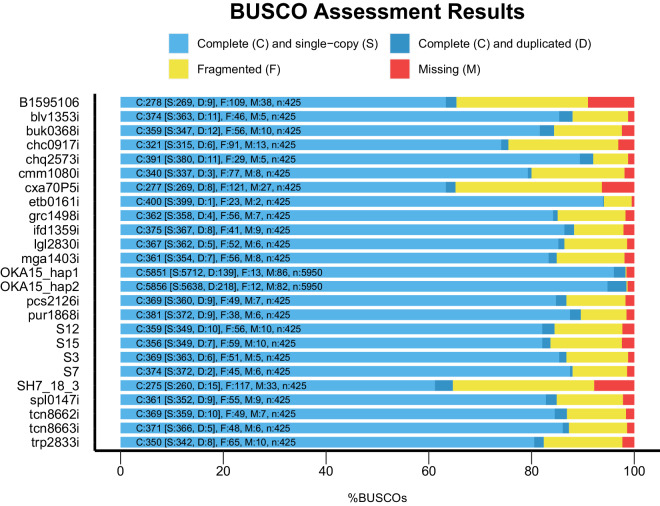
BUSCO quality assessment results of short reads *de novo* genome assemblies for 24 potato wild relatives or hybrid clones (*Solanum* sp). The bar plot shows the percent of BUSCO genes (*Viridiplantae* dataset) present in each genome assembly. Species information and accession numbers are detailed in Table 1.

Each genome was assembled into contigs from the raw Illumina reads using the *de novo* assembler *MaSuRca v4.0.5*^47^. Organelle sequences were removed from the resulting assemblies using publicly available potato plastome^48,49^ and mitogenome sequences^27,50^. The *de novo* assemblies were aligned against the organellar reference using *nucmer* from *Mummer v4.0.0beta2* utilities^34^ and the alignments were filtered for 95% identity and 200 bp alignment length. Any contigs with >95% coverage against the organelles were removed, as well as organellar sequences at the start and ends of contigs. The resulting filtered contigs were queried against the non-redundant nucleotide database using *blast*+ *v2.11.0*^51^ to remove contaminant sequences. Any contig with a reliable match (90% query converge with 90% sequence identity) to organisms outside of green plants were removed from the assembly. Finally, contigs that are less than 200 bp in length were removed and the remaining contig ids were modified to create a final clean assembly file using *BBmap v38.86*^52^ (Table 3). The plastomes of each genome were assembled and annotated from raw Illumina reads using the *Plastaumatic*^53^ pipeline. The quality of each *de novo* assembly was calculated using *QUAST v5.0.2*^54^ to determine the assembly lengths, N50 values etc, and *BUSCO v5.2.2*^55^ to check for the completeness of the assembly by looking for the presence of single copy orthologs from *Viridiplantae* (Fig. 3). An assembly was aligned against its reference genome^22^ when available using *nucmer* from *mummer v4.0.0beta2*^34^ and the alignment statistics were generated using *dnadiff*.

**Table 3:** *De novo* genome assembly statistics and the heterozygosity information.

Short ID	Species	%Heterozygosity (Max)	Estimated genome size (bp)	Final assembly size (bp)	N50	#Contigs	Largest contig (bp)
OKA15	*S. okadae*	0.88	699,974,833	728,982,852 (hap1) 725,850,496 (hap2)	58,550,880 (hap1) 58,363,667 (hap2)	214 (hap1) 131 (hap2)	84,709,123 (hap1) 83,182,665 (hap2)
B1595106	*S. brevicaule* (=*S. oplocense*) *X S. tuberosum*	11.0	671,281,508	1,007,750,739	3,547	480,556	233,393
blv1353	*S. boliviense*	0.9	656,688,793	666,810,905	23,940	66,445	286,025
buk0368	*S. bukasovii*	1.3	676,704,463	716,989,807	22,551	76,513	238,352
chc0917	*S. chacoense*	2.0	678,036,198	765,361,945	6,068	344,542	100,658
chq2573	*S. chiquidenum*	0.7	703,406,219	693,400,501	23,961	86,120	269,173
cmm1080	*S. commersonii*	1.1	735,286,606	720,550,625	17,735	105,239	183,894
cxa70P5	*S. commersonii X S. andigena*	10.0	739,885,351	957,824,389	3,340	483,218	224,152
etb0161	*S. etuberosum*	0.1	627,428,196	610,576,211	59,223	34,061	559,139
grc1498	*S. gracilifrons*	0.7	731,065,701	669,865,430	20,516	75,069	267,424
ifd1359	*S. megistacrolobum*	1.2	643,769,104	684,052,840	18,429	84,701	211,877
lgl2830	*S. lignicaule*	0.7	664,330,531	662,942,901	23,327	68,006	254,715
mga1403	*S. megistacrolobum*	1.3	659,895,487	695,075,751	18,244	82,893	188,119
pcs2126	*S. paucissectum*	1.1	725,955,590	734,795,577	20,376	96,165	234,965
pur1868	*S. piurae*	0.7	741,119,656	720,192,443	23,712	84,700	248,207
S12	*S. tarijense*	1.3	681,481,062	711,715,600	19,861	87,536	236,814
S15	*S. gandarillasii*	1.0	684,676,028	686,228,213	20,834	84,293	308,125
S3	*S. commersonii*	0.7	678,163,656	660,741,360	23,691	62,463	238,718
S7	*S. pinnatisectum*	1.5	575,316,904	619,463,363	28,631	49,365	339,432
SH7_18_3	*S. tarnii X S. tuberosum*	10.3	795,104,822	1,069,548,688	3,328	548,546	189,557
spl0147	*S. sparsipilum*	1.3	658,080,483	697,910,295	21,584	76,602	190,640
tcn8662	*S. tacnaense*	1.0	693,678,525	714,416,403	20,975	77,730	177,444
tcn8663	*S. tacnaense*	1.1	694,313,399	728,989,011	22,321	75,214	237,438
trp2833	*S. tarapatanum*	1.3	724,172,909	717,717,986	15,266	98,790	164,540

**Fig. 4:**
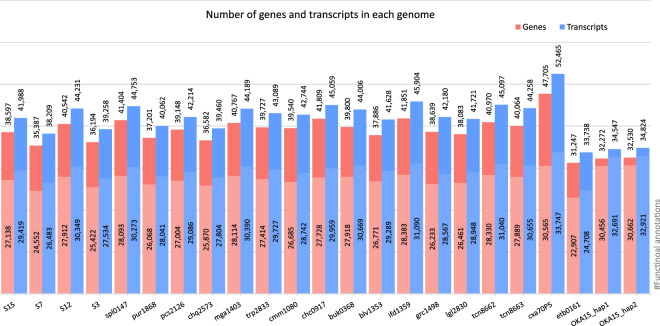
The number of high-confidence genes and transcripts found in 22 potato wild relatives accessions (*Solanum* sp). The two columns for each genome represent the total number of predicted genes and transcripts (larger number on top of bar) along with their proportions of functional annotations (smaller number inside bar). Species information and accession numbers are detailed in Table 1.

The genome assemblies were annotated using the RNA-Seq data as the evidence for gene prediction along with homology-based prediction. The two accessions (B1595106 and SH7_18_3) with no RNA-Seq data were annotated only with the protein sequences. A repeat masking was performed on each genome assembly using *RepeatMasker v4.1.2-pl*^37^ with a custom repeat library of potato reference sequences constructed by concatenating repeat libraries from the *Petota* super pangenome^26^ and DMv6.1^9^. Table 4 details the number of bases masked in each genome and size of different types of transposable elements found in them. Then the structural annotation was performed using *BRAKER v2.1.6*^40,56,57^ in two runs, one with the RNA-Seq data as evidence and another run with protein sequences as evidence. The trimmed RNA-Seq reads of each accession were aligned to its genome assembly using *HiSAT2 v2.2.1*^39^ for BRAKER1^57^. Most of the accessions have an optimal RNA-Seq alignment rate (>65%) against their short read *de novo* assemblies. The alignment files were sorted and indexed using *SAMtools v1.16.1*^31^. Then the structural annotation was performed using the BRAKER pipeline^58–63^ on the masked genome assembly with the alignment file. The BRAKER pipeline performed gene prediction by *AUGUSTUS v3.4.0*^64^ and *GeneMark-ET*^65,66^ to generate gene structural annotations. For the homology-based prediction (*BRAKER2*), *Solanales* protein sequences from *OrthoDB* along with the protein sequences from potato reference genomes^9,18,67^ were combined to create a protein database for *BRAKER*. The *BRAKER1* and *BRAKER2* results were combined to select transcripts based on support by extrinsic evidence using *TSEBRA v1.0.3*^68^ to get a high-confidence gene set. The annotations for the accessions with no RNA-Seq data (B1595106 and SH7_18_3) were obtained from the *BRAKER2* run with protein sequences as evidence. Since no RNA-Seq evidence was available for these two, all the predicted structural genes were added to the final gene set. Figure 4 shows the number of genes and transcripts predicted in these accessions by the *BRAKER* pipeline. A total of 31,247–47,705 high confidence genes (i.e. genes with RNA-Seq evidence) were found in these genomes, with *S. etuberosum* having the lowest number of genes and the S*. commersonii* X *S. andigena* hybrid having the greatest number of genes. A high confidence gene set was not generated for B1595106 and SH7_18_3 genomes due to lack of the RNA-Seq data. A functional annotation was performed using *Interproscan v5.52-86*^42^ against the PFAM database. Approximately 65–70% of the annotated genes in all the genomes were found to have PFAM domains, except for the SH7_18_3, and B1595106 genomes (for which RNA-Seq data was not available).

**Table 4:** Results of the repeat analysis with different classes of TEs and their size.

Short ID	Species	Size (bp)	Bases masked (bp)	LTR elements (length occupied)	SINEs (length occupied)	LINEs (length occupied)	DNA TE (length occupied)	Small RNA
oka15	*S. okadae*	1,454,833,348	916,145,551	382,327,733	311,969	31,610,488	27,977,851	8,910,891
B1595106	*S. brevicaule (*=*S. oplocense) X S. tuberosum*	1,007,750,739	701,616,563	350,301,794	1,149,341	21,372,177	27,305,055	1,460,888
blv1353	*S. boliviense*	666,810,905	435,060,802	208,807,085	869,239	16,084,967	21,192,414	990,869
buk0368	*S. bukasovii*	716,989,807	480,158,888	235,333,428	848,893	17,276,972	20,948,217	1,046,511
chc0917	*S. chacoense*	765,361,945	513,233,181	243,991,092	954,158	17,069,642	23,354,232	1,076,340
chq2573	*S. chiquidenum*	693,400,501	466,348,914	223,602,249	768,785	16,231,519	20,580,712	940,668
cmm1080	*S. commersonii*	720,550,625	485,952,044	243,982,820	814,492	16,889,446	22,470,598	1,067,960
cxa70P5	*S. commersonii X S. andigena*	957,824,389	653,608,360	315,996,868	1,157,382	20,509,112	26,519,625	1,559,458
etb0161	*S. etuberosum*	610,576,211	407,532,810	215,984,646	695,999	13,262,589	11,258,462	1,037,743
grc1498	*S. gracilifrons*	669,865,430	444,824,908	216,390,612	826,044	15,725,904	20,096,452	1,035,345
ifd1359	*S. megistacrolobum*	684,052,840	444,642,488	211,138,196	890,857	16,205,334	20,757,278	1,033,001
lgl2830	*S. lignicaule*	662,942,901	435,332,101	210,672,488	851,994	16,142,336	20,366,503	935,951
mga1403	*S. megistacrolobum*	695,075,751	456,966,566	220,900,148	862,821	16,633,307	20,828,617	1,129,864
pcs2126	*S. paucissectum*	734,795,577	499,861,459	244,892,952	808,303	17,179,519	21,268,375	933,269
pur1868	*S. piurae*	720,192,443	491,426,301	242,004,208	781,453	16,664,457	20,933,611	921,302
S12	*S. tarijense*	711,715,600	470,513,861	226,540,751	884,250	16,607,481	22,555,724	1,044,403
S15	*S. gandarillasii*	686,228,213	451,836,344	215,877,305	865,194	16,770,897	21,598,771	981,756
S3	*S. commersonii*	660,741,360	434,370,170	214,113,765	783,717	16,270,941	21,546,608	923,547
S7	*S. pinnatisectum*	619,463,363	401,435,659	195,095,305	743,115	15,709,814	18,201,493	910,108
SH7_18_3	*S. tarnii X S. tuberosum*	1,069,548,688	738,444,227	363,846,598	1,246,886	23,381,848	29,029,059	1,560,804
spl0147	*S. sparsipilum*	697,909,412	457,896,220	218,926,860	883,367	16,834,545	22,095,633	992,710
tcn8662	*S. tacnaense*	714,416,403	474,138,047	230,699,916	882,481	17,044,624	21,513,973	1,203,905
tcn8663	*S. tacnaense*	728,989,011	489,162,880	242,451,878	871,253	17,339,593	21,361,744	1,024,620
trp2833	*S. tarapatanum*	717,717,986	478,288,796	235,624,996	866,639	16,996,387	21,120,093	969,990

### Presence/absence variation analysis

**Fig. 5:**
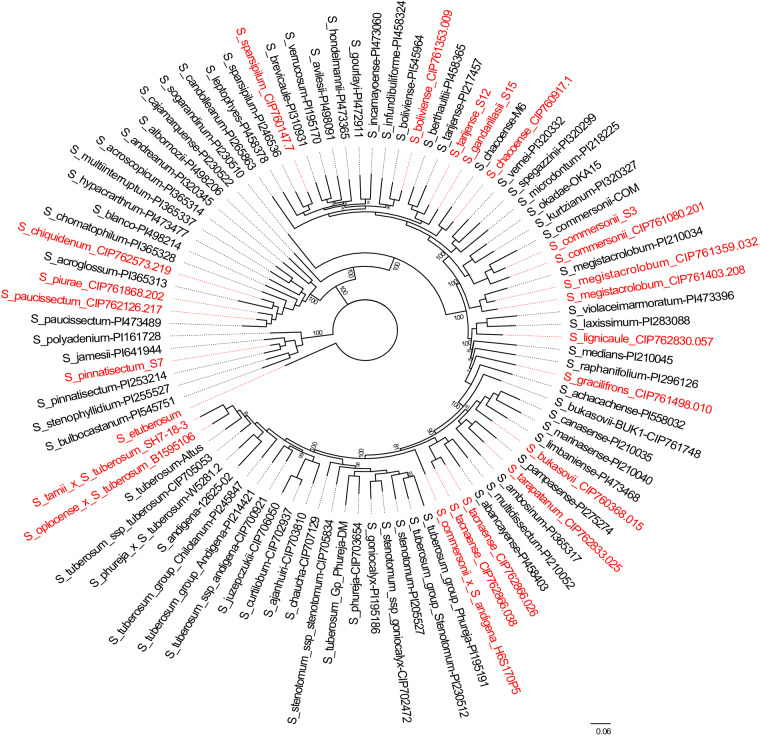
A whole genome presence-absence variation (PAV) based phylogenetic tree showing the relatedness of potato wild relatives (*Solanum* sp). The *S. etuberosum* genome sequence was used as an outgroup. The numbers at the nodes represent bootstrap support values. For clarity of the figure, only key values are given, and the ones omitted are all 100.

**Fig. 6:**
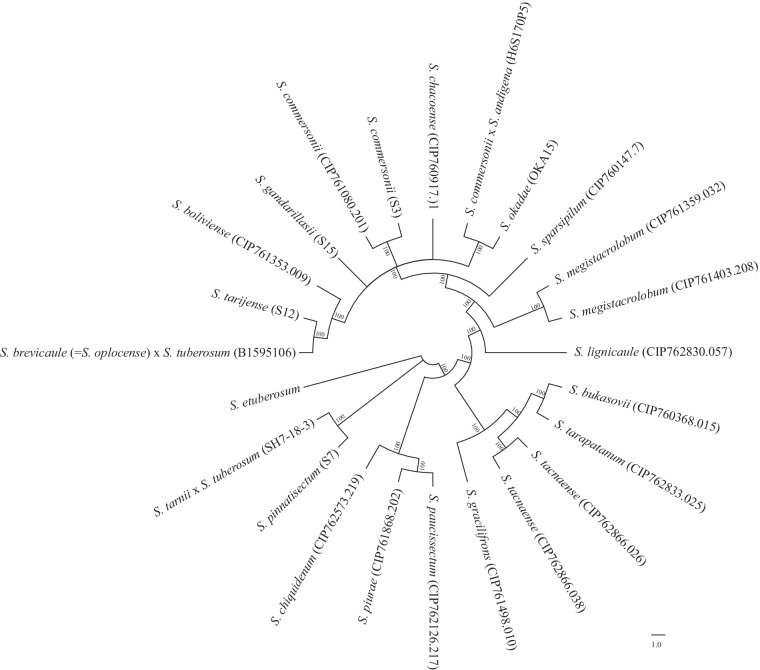
A plastome sequence based phylogenetic tree of 24 potato wild relatives (*Solanum* sp). The *S. etuberosum* was used as an outgroup. The numbers at the nodes represent bootstrap support values.

The trimmed reads of each genome were aligned to the *Petota* super pangenome^26^ using *bwa v0.7.17*^69^. The resulting alignments were filtered to keep only properly paired (*-f 2*) and remove secondary alignments (*-F 2048*), then sorted and indexed using *SAMtools v1.13*^31^. The gene presence/absence variations (PAVs) were identified in each genome using *SGSGeneLoss v0.1*^70^ with *minCov* = *2* and *lostCutoff* = *0.2*. These PAVs were used to generate a maximum-likelihood phylogenetic tree using *IQ-TREE v2.1.3*^71^ with GTR2 + FO + R4 as a substitution model and 1000 bootstrap replicates and *S. etuberosum* set as an outgroup (Fig. 5). A multiple sequence alignment of the 23 plastomes was made using *MAFFT v7*^72^, followed by a plastome based parsimonious phylogenetic tree constructed using *paup v4.0a*^73^ with 1000 bootstrap replicates and *S. etuberosum* as an outgroup (Fig. 6). The phylogenetic trees were visualized in *FigTree v1.4.4* (http://tree.bio.ed.ac.uk/software/figtree/).

## Data Records

*S. okadae* (OKA 15) genome sequence data is deposited at NCBI under BioProject PRJNA684565^74^ and BioSample SAMN17860560^75^**:** the Hi-C data under SRR26081972^76^; Nanopore SRR20870051^77^; PacBio SRR20870052^78^; Illumina reads SRR14482384^79^; and the phased haplotype genome assemblies are available under BioProject PRJNA1018115^80^ (haplotype 1) JAWDCX000000000^81^ and PRJNA1018115^82^ (haplotype 2) JAWDCY000000000^83^. The OKA 15 RNA-Seq data BioSamples are available under SAMN37429684- SAMN37429686^84–86^ and the RNA-Seq Illumina reads are available under SRR26082554-SRR26082556^87–89^.

All other data used in this study is deposited in NCBI and available under the BioProject PRJNA779368^90^. The sample descriptions of the 23 genomes used in the genome sequencing are available under the BioSample accession numbers (SAMN23440977^91^, SAMN23440980 – SAMN23440983^92–95^, SAMN23440986 – SAMN23440990^96–100^, SAMN23440993 – SAMN23440998^101–106^, SAMN23441000 – SAMN23441002^107–109^, SAMN23441004 – SAMN23441007^110–113^). The Illumina WGS reads of each genome were deposited with their respective Biosample IDs under the Sequence Read Archive (SRA) submission (SRR17078416 – SRR17078418^114–116^, SRR17078420^117^, SRR17078422 – SRR17078424^118–120^, SRR17078426 – SRR17078432^121–127^, SRR17078435 – SRR17078439^128–132^, SRR17078441 – SRR17078444^133–136^). The final genome assemblies were deposited under the WGS assembly projects with the accession numbers JAJONR000000000 – JAJONU000000000^137–139^, JAJONW000000000 – JAJOOD000000000^140–148^, JAJOOF000000000 – JAJOOJ000000000^149–153^, JAJOOM000000000 – JAJOOP000000000^154–157^, JAJOOS000000000^158^, JAKRZT000000000^159^ and the 23 plastome sequences were deposited in the GenBank database with the accession numbers OM638053-OM638057^160–164^, OM638059-OM638064^165–170^, OM638066-OM638068^171–173^, OM638071-OM638072^174,175^, OM638074-OM638077^176–179^, OM638081-OM638083^180–182^. A complete list of WGS raw data and assemblies are available in Table 5. The sample descriptions of the RNA-Seq samples are available under the BioSample accession numbers SAMN32886741 – SAMN32886746^183–188^, SAMN32886748 – SAMN32886751^189–192^, SAMN32886756 – SAMN32886763^193–200^, SAMN32886766 – SAMN32886771^201–206^, SAMN32886782 – SAMN32886784^207–209^, SAMN32886786^210^, SAMN32886787^211^, SAMN32886790 – SAMN32886796^212–218^, SAMN32886798^219^, SAMN32886799^220^ and their raw sequencing reads are available under the SRA accession numbers SRR23225904 - SRR23225909^221–226^, SRR23225913^227^, SRR23225914^228^, SRR23225916 – SRR23225918^229–231^, SRR23225929^232^, SRR23225930^233^, SRR23225933^234^, SRR23225938 – SRR23225940^235–237^, SRR23225942 – SRR23225944^238–240^, SRR23225947 – SRR23225951^241–245^, SRR23225953 – SRR23225955^246–248^, SRR23225960 – SRR23225962^249–251^, SRR23225964^252^, SRR23225966 – SRR23225971^253–258^ (Table 6). The gene annotations are available at the following link: https://potatogenomeportal.org/download

**Table 5:** A detailed list of genomic data descriptor records. It includes BioSample, SRA, genome, and plastome assembly accession numbers of each genome.

Short ID	Species	BioSample	Nuclear genome assembly	WGS reads	Plastome
oka14	*S. okadae*	SAMN17860560	JAWDCX000000000/JAWDCY000000000	SRR26081972 (Hi-C) SRR20870051 (Nanopore) SRR20870052 (PacBio) SRR14482384 (10X)	MW592006
chq2573	*S. chiquidenum*	SAMN23440977	JAJOOS000000000	SRR17078430	OM638057
chc0917	*S. chacoense*	SAMN23440980	JAJOOP000000000	SRR17078444	OM638056
pur1868	*S. piurae*	SAMN23440981	JAJOOO000000000	SRR17078443	OM638068
mga1403	*S. megistacrolobum*	SAMN23440982	JAJOON000000000	SRR17078442	OM638066
cmm1080	*S. commersonii*	SAMN23440983	JAJOOM000000000	SRR17078441	OM638059
blv1353	*S. boliviense*	SAMN23440986	JAJOOJ000000000	SRR17078439	OM638054
buk0368	*S. bukasovii*	SAMN23440987	JAJOOI000000000	SRR17078438	OM638055
grc1498	*S. gracilifrons*	SAMN23440988	JAJOOH000000000	SRR17078437	OM638062
ifd1359	*S. megistacrolobum*	SAMN23440989	JAJOOG000000000	SRR17078436	OM638063
lgl2830	*S. lignicaule*	SAMN23440990	JAJOOF000000000	SRR17078435	OM638064
tcn8663	*S. tacnaense*	SAMN23440993	JAJOOD000000000	SRR17078432	OM638082
tcn8662	*S. tacnaense*	SAMN23440994	JAJOOC000000000	SRR17078431	OM638081
trp2833	*S. tarapatanum*	SAMN23440995	JAJOOB000000000	SRR17078429	OM638083
cxa70P5	*S. commersonii X S. andigena*	SAMN23440996	JAJOOA000000000	SRR17078428	OM638060
pcs2126	*S. paucissectum*	SAMN23440997	JAJONZ000000000	SRR17078427	OM638067
spl0147	*S. sparsipilum*	SAMN23440998	JAKRZT000000000	SRR17078426	OM638077
S15	*S. gandarillasii*	SAMN23441000	JAJONY000000000	SRR17078424	OM638072
S7	*S. pinnatisectum*	SAMN23441001	JAJONX000000000	SRR17078423	OM638075
S12	*S. tarijense*	SAMN23441002	JAJONW000000000	SRR17078422	OM638071
S3	*S. commersonii*	SAMN23441004	JAJONU000000000	SRR17078420	OM638074
B1595106	*S. brevicaule* ( = *S. oplocense*) *X S. tuberosum*	SAMN23441005	JAJONT000000000	SRR17078418	OM638053
SH7-18-3	*S. tarnii X S. tuberosum*	SAMN23441006	JAJONS000000000	SRR17078417	OM638076
etb0161	*S. etuberosum*	SAMN23441007	JAJONR000000000	SRR17078416	OM638061

**Table 6:** A detailed list of transcriptomic data descriptor records. It includes BioSample and their respective SRA accession numbers of individual samples.

Short ID	Species	SRA accession	BioSample accession	BioSample name
oka15	*S. okadae*	SRR26082556	SAMN37429684	OKA15_leaf
oka15	S. *okadae*	SRR26082555	SAMN37429685	OKA15_sprout
oka15	*S. okadae*	SRR26082554	SAMN37429686	OKA15_tuber
chq2573	*S. chiquidenum*	SRR23225967	SAMN32886745	chq2573_Leaf
chq2573	*S. chiquidenum*	SRR23225966	SAMN32886746	chq2573_Shoot
chc0917	*S. chacoense*	SRR23225970	SAMN32886741	chc0917_Flower
chc0917	*S. chacoense*	SRR23225969	SAMN32886742	chc0917_Leaf
chc0917	*S. chacoense*	SRR23225968	SAMN32886743	chc0917_Shoot
chc0917	*S. chacoense*	SRR23225971	SAMN32886744	chc0917_Tuber
pur1868	*S. piurae*	SRR23225948	SAMN32886762	pur1868_Leaf
pur1868	*S. piurae*	SRR23225947	SAMN32886763	pur1868_Shoot
mga1403	*S. megistacrolobum*	SRR23225955	SAMN32886756	mga1403_Flower
mga1403	*S. megistacrolobum*	SRR23225954	SAMN32886757	mga1403_Leaf
mga1403	*S. megistacrolobum*	SRR23225953	SAMN32886758	mga1403_Shoot
cmm1080	*S. commersonii*	SRR23225964	SAMN32886748	cmm1080_Leaf
cmm1080	*S. commersonii*	SRR23225962	SAMN32886749	cmm1080_Shoot
cmm1080	*S. commersonii*	SRR23225961	SAMN32886750	cmm1080_Tuber
blv1353	*S. boliviense*	SRR23225907	SAMN32886792	blv1353_Leaf
buk0368	*S. bukasovii*	SRR23225906	SAMN32886793	buk0368_Leaf
grc1498	*S. gracilifrons*	SRR23225905	SAMN32886794	grc1498_Leaf
ifd1359	*S. megistacrolobum*	SRR23225904	SAMN32886795	ifd1359_Leaf
lgl2830	*S. lignicaule*	SRR23225933	SAMN32886796	lgl2830_Leaf
tcn8663	*S. tacnaense*	SRR23225930	SAMN32886798	tcn8662_Leaf
tcn8662	*S. tacnaense*	SRR23225929	SAMN32886799	tcn8663_Leaf
trp2833	*S. tarapatanum*	SRR23225918	SAMN32886782	trp2833_Flower
trp2833	*S. tarapatanum*	SRR23225917	SAMN32886783	trp2833_Leaf
trp2833	*S. tarapatanum*	SRR23225916	SAMN32886784	trp2833_Shoot
cxa70P5	*S. commersonii X S. andigena*	SRR23225960	SAMN32886751	cxa70P5_Shoot
pcs2126	*S. paucissectum*	SRR23225951	SAMN32886759	pcs2126_Leaf
pcs2126	*S. paucissectum*	SRR23225950	SAMN32886760	pcs2126_Shoot
spl0147	*S. sparsipilum*	SRR23225939	SAMN32886770	spl0147_Leaf
spl0147	*S. sparsipilum*	SRR23225938	SAMN32886771	spl0147_Shoot
S15	*S. gandarillasii*	SRR23225944	SAMN32886766	S15_Leaf
S15	*S. gandarillasii*	SRR23225943	SAMN32886767	S15_Tuber
S7	*S. pinnatisectum*	SRR23225942	SAMN32886768	S7_Leaf
S7	*S. pinnatisectum*	SRR23225940	SAMN32886769	S7_Tuber
S12	*S. tarijense*	SRR23225914	SAMN32886786	S12_Leaf
S12	*S. tarijense*	SRR23225913	SAMN32886787	S12_Tuber
S3	*S. commersonii*	SRR23225909	SAMN32886790	S3_Leaf
S3	*S. commersonii*	SRR23225908	SAMN32886791	S3_Tuber
etb0161	*S. etuberosum*	SRR23225949	SAMN32886761	PI245939_Leaf

## Technical Validation

### Sequencing data quality control

The quality of the raw WGS and RNA-Seq reads were checked using *FastQC*^43^. Various metrics such as per base sequence quality, overall read quality score, GC content, N content, and overrepresented sequences were analyzed to detect low quality sequences or biases in the data. We have found no issues or bias in the WGS reads, whereas the RNA-Seq data is found to have overrepresented sequences in some of the datasets, which is not unusual for RNA-Seq data. We have also checked for viral genome sequences in the RNA-Seq data since potato plants are frequently found to have various types of potato viruses. The reads were classified using *kraken2 v2.1.2*^38^ by searching against their own genome assemblies and potato virus sequences downloaded from NCBI. Most genomes have very few hits to the viral sequences, and we removed reads that have hits to viruses from each sample and used the clean reads in the following analyses.

### Quality assessment of the genome assemblies

The *de novo* genome assembly quality was assessed by *QUAST*^54^ and *BUSCO*^55^ where contiguity and completeness of the assemblies were analyzed. The OKA15 genome assembly haplotype 1 and 2 have a complete BUSCO score of 98.3% and 98.5%, respectively, when compared to the (solanales_odb10). *Merqury*^259^ was used to estimate the completeness (QV). The complete OKA15 assembly was 99.4%, while haplotype 1 and haplotype 2 were 65 and 65% complete individually (meaning 15–30% of the reads map equally well to both haplotypes). Haplotype 1 (hap1) has 214 contigs with a total length of 729 Mb. The largest contig is 84.7 Mb and the N50 is 58.6 Mb. Haplotype 2 (hap2) has 131 contigs with a total length of 726 Mb, the largest contig is 83.2 Mb and the N50 58.4 Mb. The heterozygosity of the oka15 genome was calculated using *jellyfish*^45^ and found to be 0.87%.

The *S. etuberosum* genome, which has been self-pollinated for several generations, has the best assembly of all with an N50 value of 59,223, 34,061 contigs, and 610 Mbp genome size. The three tetraploids (B1595106, SH7_18_3, and cxa70P5), and a diploid *S. chacoense* (chc0917) have fragmented assemblies (Table 2). Nine genome assemblies from this study were also compared with long-read assemblies of the same species from a recent study^22^. Overall, the amount of sequences that mapped to their individual reference ranged from ~85–99% with ~79–98% of the assembly coverage (Table 7). The BUSCO assessment of the assemblies revealed the majority of them are complete with presence of >80% complete genes (Fig. 2). Overall, the *S. etuberosum* genome has the highest percent of core plant orthologous genes with 94.1% of genes present as complete copies, followed by the chq762573 and pur1868 genomes with 92% and 89.6% of complete BUSCO genes. The SH7_18_3, cxa70P5, and B1595106 genomes have a higher percentage of fragmented genes among the 23 genomes. High rates of heterozygosity and higher ploidy levels are the major contributing factors to highly fragmented assemblies^38^.

**Table 7:** Alignment statistics of nine genomes from this study compared against reference genomes.

Short ID	Species	Reference	% Aligned Seqs	% Aligned Bases	Avg Identity	SNPs
etb0161	*S. etuberosum*	*S. etuberosum* (PG0019)	96.74	97.79	98.47	5,125,086
S7	*S. pinnatisectum*	*S. pinnatisectum* (PG1013)	95.06	96.37	98.71	4,034,460
pcs2126	*S. paucissectum*	*S. paucissectum* (PG3022)	84.83	78.76	91.98	16,608,649
pur1868	*S. piurae*	*S. piurae* (PG3023)	86.62	84.15	94.06	15,723,546
lgl2830	*S. lignicaule*	*S. lignicaule* (PG4017)	96.82	97.17	99.19	2,848,239
chc0917	*S. chacoense*	*S. chacoense* (PG4042)	97.79	94.8	97.51	9,402,305
cmm1080	*S. commersonii*	*S. commersonii* (PG4049)	98.96	97.73	98.6	5,454,247
S3	*S. commersonii*	*S. commersonii* (PG4049)	98.39	95.18	96.97	10,870,964
blv1353	*S. boliviense*	*S. boliviense* (PG5076)	96.88	88.4	94.95	14,093,976

### Phylogenetic inference

Previous studies classified section *Petota* (tuber-bearing) into major Clades and subgroups^13,26,260,261^. Here we have reconstructed phylogenetic trees from the PAV data (Fig. 5) as well as plastome sequences (Fig. 6) to understand the relationship between these genomes. The results have shown similar groupings as previous studies where *S. pinnatisectum* (Clade 1 + 2), *S. paucissectum*, *S. piurae*, and *S. chiquidenum* (Clade 3), and the remaining accessions (Clade 4) separated into different clades. Within Clade 4, the *S. megistacrolobum* accessions formed a sister clade to Clade 4 South, something which was also seen in the *Petota* pangenome^26^. *S. okadae* placed in the Clade 4 South as also seen in the *Petota* pangenome. The *S. tarnii X S. tuberosum* (SH7_18_3) genome, which is a somatic hybrid between *S. tarnii* (Clade 1 + 2 species) and *S. tuberosum* (Clade 4) obtained by *in vitro* fusions of protoplasts, placed differently in the PAV and plastome phylogenetic trees. In the plastome phylogenetic tree, it is grouped with the Clade 1 + 2 species, whereas, in the PAV tree it is grouped with another *S. tuberosum* hybrid (Clade 4) showing the phylogenetic difficulty with hybrids and the importance of including both nuclear and organellar data. Moreover, genomes that are known to be closely related are grouped together, and genomes representing the same species grouped together, which validates the use of PAVs in determining phylogenetic relationships^26^.

## Usage Notes

The *Solanum* section *Petota* has over 100 cultivated and wild species. The potato crop wild relatives are an excellent source of genetic variation; however, not many sequencing efforts have been carried out to study these wild species. Here, we present the 24 phased chromosomes for *S. okadae*, and genome and transcriptome data for an additional 23 potato wild relative accessions. Though most of these are short-read assemblies, they are a useful tool for exploring genetic diversity that can provide insight in potato pre-breeding. The WGS reads can be used in various analyses such as determining SNPs, structural variations, and PAVs to understand the genetic diversity that exists within the section *Petota*. The RNA-Seq data of these wild species with important agronomic traits is especially crucial to understand their transcriptome profile. *S. okadae* foliage has been shown to contain the glycoalkaloid tomatine, and undetectable levels of solanine and chaconine, and carries Colorado potato beetle resistance, drought and cold tolerance (but not frost) and moderate late blight resistance^262–265^.

## Data Availability

All software with their specific version used for data processing are clearly described in the methods section. If no specific variable or parameters are mentioned for a software, the default parameters were used.
